# Urban schistosomiasis and associated determinant factors among school children in Bamako, Mali, West Africa

**DOI:** 10.1186/2049-9957-4-4

**Published:** 2015-01-29

**Authors:** Abdoulaye Dabo, Adama Z Diarra, Vanessa Machault, Ousmane Touré, Diarra Sira Niambélé, Abdoulaye Kanté, Abdoulaye Ongoiba, Ogobara Doumbo

**Affiliations:** Department of Epidemiology of Infectious Diseases, Faculty of Medicine, Pharmacy and Dentistry, University of Techniques and Technologies of Bamako, Box 1805, Bamako, UMI 3189 Mali; Unité d’entomologie médicale, Equipe 7, Maladies émergentes et moustiques, Institut de Médecine Tropicale du Service de Santé des Armées, Allée du Médecin Colonel Jamot, Parc du Pharo, BP60109, 13262 Marseille Cedex 07, France

**Keywords:** Schistosomiasis, Snails, Breeding sites, Endemization, Bamako, Mali

## Abstract

**Background:**

Schistosomiasis is classically described as a rural disease that occurs in areas with poor sanitary conditions. However, over recent decades, there has been an expansion of schistosomiasis foci towards urban areas faced with a rapid and disordered urbanization. In Bamako, Mali, the impact of environmental change on vector-borne diseases such as schistosomiasis is not well known. This study sought to identify the presence of schistosomiasis transmission hotspots in Bamako. Using this perspective, we aimed to describe the risk factors of the endemization and maintenance of schistosomiasis.

**Materials and methods:**

A cross-sectional study was carried out in the six municipalities (communes) in Bamako. Environmental information was obtained from earth observation satellites in order to maximize ecological contrasts. Twenty-nine blocks of 200 m x 200 m were identified. We selected a school inside or nearest to each block for urine and stool samples examination. The study cohort was school children aged between eight and 15 years. The Kato-Katz technique and filtration were used for *Schistosoma mansoni* and *S. haematobium* ova research in stools and urine, respectively. The schools and snail breeding sites were georeferenced. Four malacological surveys were conducted between October 2011 and February 2012. Bivariate analysis was used to identify independent predictors of being infected with schistosomiasis.

**Results:**

The prevalence rate of *S. haematobium* was 14.7% (n = 1,761) and that of *S. mansoni* 1.5% (n = 1,491). Overall, the urinary form was endemic in 76.6% of schools. The infection significantly varied between the municipalities (*p < 0.001*). It was also more prevalent on the left side of the Niger River than the right side (17.4% vs. 9.5% respectively; *p < 0.001*). The vicinity to snail breeding sites (OR = 3.677; 95% IC [2.765–4.889]; *p < 10*^*-3*^) and parents’ occupations (OR = 7.647; 95% IC [2.406–24.305]; *p < 0.001*) were the most important risk factors associated with *S. haematobium* infection exposure. *Biomphalaria pfeifferi*, *Bulinus truncatus*, and *B. globosus* were the intermediate hosts captured. The schistosome natural infection rates (SNIRs), which were low or nil in October and November, rose to 2.8% in January and 8.3% in February for *B. pfeifferi* and *B. truncatus*, respectively.

**Conclusion:**

Our findings show that there is a high transmission risk for schistosomiasis in Bamako. Appropriate integrated control measures need to be introduced to control the transmission of this disease in the study area.

**Electronic supplementary material:**

The online version of this article (doi:10.1186/2049-9957-4-4) contains supplementary material, which is available to authorized users.

## Multilingual abstracts

Please see Additional file 1 Multilingual abstracts in the six official working languages of the United Nations.

## Background

Schistosomiasis is the second most prevalent tropical water-borne disease after malaria, and a leading cause of severe morbidity in many parts of the world. According to a recent estimate, 207 million people were infected by mid-2003. Active transmission is reported in 67 countries, of which 46 are in Africa [1]. In relation to transmission conditions, schistosomiasis is classically described as a rural disease that occurs in areas without potable water and adequate sanitation. Nevertheless, because of migration, and rapid and disordered urbanization, urban areas in Africa and South America are now foci of transmission. In Africa, the disease is endemic in cities such as Ibadan (Nigeria) [2], Addis Ababa (Ethiopia) [3], and Kisumu (Kenya) [4]. One of the major factors noted in the process of “urbanization” of schistosomiasis is the migratory flow of the infected rural population, which occurs due to more attractive employment opportunities in the urban areas [5].

Infections due to *Schistosoma haematobium* and *S. mansoni* have existed in Bamako since 1953 [6]. From this period up until now, many surveys have been conducted but they have been geographically limited to one or two quarters [7, 8]. Data collected on disease and snail intermediate hosts were punctual but incomplete, and didn’t reflect the epidemiology of infection in all districts of Bamako. Knowing information on global rates of schistosomiasis and snail distribution could help to identify hotspots in the city and could lead to treatment been given to those who need it most (in terms of prevalence and intensity of infection). It is crucial to describe the risk factors that give rise to occurrences of schistosomiasis in order to identify the mechanisms for transmission and maintenance of this endemic disease in Bamako.

## Methods

### Study area

**Figure 1:**
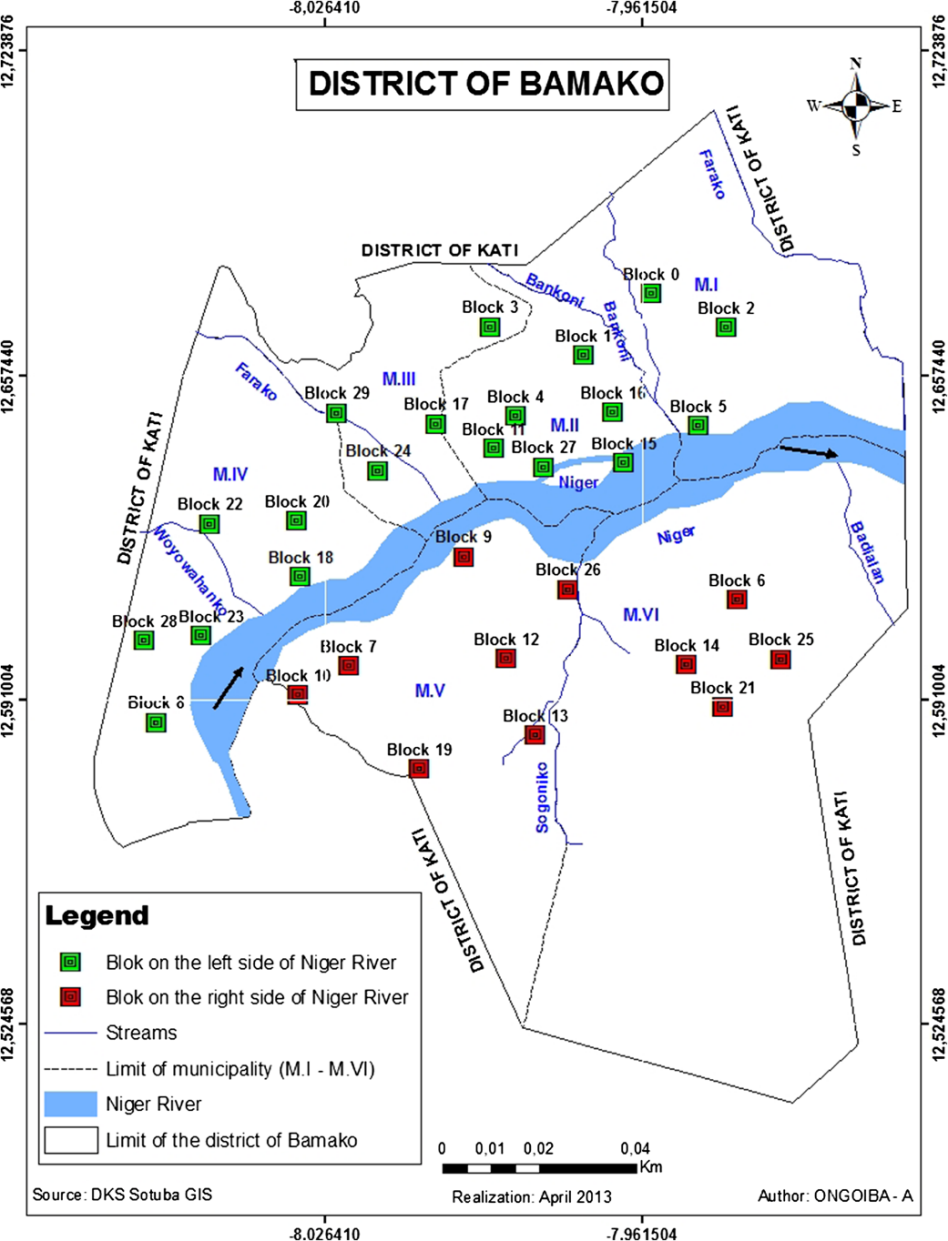
**Map of Bamako showing the localization of studied blocks.**

The study was conducted in Bamako (12°39’ N latitude and 8°0’ W longitude), the capital city of Mali (see Figure 1). The surface area of the city is 1420 km^2^. The town looks like a big basin, surrounded in part by hills, with the Niger River and its tributaries flowing across. The town belongs to the North-Soudanian climatic zone with two major seasons: the wet season from November to May with its beginning and end marked by torrential rains and thunderstorms, and the dry season from April to October. The mean annual rainfall is about 1,400 mm, which occurs mainly during the period from July to September. Temperatures are generally high and almost uniform throughout the year with a mean annual maximum temperature of 33°C and a mean annual minimum temperature of 22°C [9]. The tributaries of the Niger River must be used to collect water from the rain but they have been turned into a refuse dump, which leads to a slow flow or stagnant water, and in turn makes them suitable breeding sites of snail intermediate hosts.

Bamako was founded at the end of the 16^th^ century. In 2009, the population was 1,809,106, with an annual growth of 4.8% [9]. Unfortunately, this rapid and disordered growth has not been followed by improved sanitation, sewage systems, and the right water supplies. The city is partitioned into six municipalities, ranging from M-I to M-VI and more than 50 quarters (see Figure 1). There are four municipalities (I–IV) on the left side and two (V–VI) on the right side of the Niger River. There are about 736,183 inhabitants on the left side compared to 849,727 on the right side. People first occupied the left side, especially the quarter of Niaréla in the CII, near the River. Progressively, and in accordance with the city’s growth due to various factors including migration, other quarters appeared along the Niger River and its tributaries. The quarters on the left side of the River (also named the old town) are characterized by high population densities and poor sanitation conditions compared to those on the right side, which are more spaced out and located further from the River. The municipalities on the right side are newer as they were established after the independence of the country in 1960. The town presents a central zone including the municipalities II, III, and one part of the M-IV called ACI 2000, which contains most of the administrative offices and main industries, and is marked by a high population density.

### Study design and sampling techniques

The survey was conducted in twenty-nine (29) blocks, each 200 m × 200 m, in Bamako. These blocks were selected on the basis of the images from the SPOT-5 (Satellite Pour l’Observation de la Terre), part of the National Aeronautics and Space Administration’s (NASA) Earth Observing System [10], at 2.5 m of spatial resolution obtained on March 2, 2009. The non-supervised classification method of the image allows the generation of an earth occupation map. The 29 blocks were selected in order to maximize the ecological (the presence of a permanent water collection system), environmental (how the population manages garbage, and stool and urine residues), and anthropogenic (the relationship between population and water collection) contrasts. According to the distribution of the blocks through the municipalities, three blocks in the M-I, six in the M-II, four in the M-III, five in the M-IV, seven in the M-V, and four in the M-VI were selected. Once the blocks were chosen definitively, a precise mapping was done after collecting GPS points around them.

One school inside or nearest to each of the 29 blocks was chosen for parasitological investigations in order to study the distribution of schistosomes in Bamako. Similarly, all the human water contact sites in the Niger River or its tributaries located near the chosen schools were observed for snail breeding sites.

**Figure 2:**
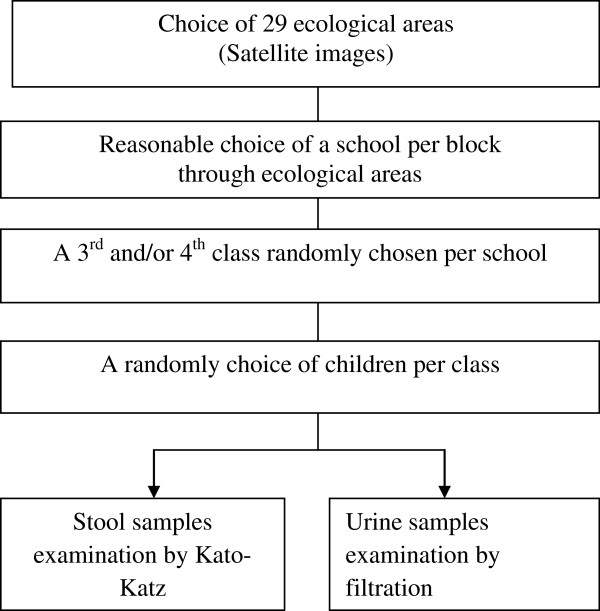
**Diagram for selection of block, schools and study population in the district of Bamako, January 2011.**

According to the World Health Organization (WHO) guidelines, 200 to 250 individuals (50 per block or school) from third or fourth year primary school classes (9–10-year-old schoolchildren) should be an adequate sample for an ecologically homogeneous area in order to evaluate prevalence and intensity [11]. The minimum sample size was thus 1,450 (29 blocks × 50). We added 10% (n = 145) to this sample size for the loss of view (n = 1,595). In each school, the children were randomly selected using the class rosters. Figure 2 shows the selection of blocks, schools, classes, and samples for urine and stool examination.

### Malacological survey

The survey sites were selected on the basis of their water contact points, that is where people consistently go to collect water, wash clothes, bathe, swim, play (young children), and wash cars. Snail sampling was conducted from October 2011 to February 2012on these major water contact sites on the Niger River or its tributaries. During snail collection, observations were also made on the physical characteristics of the habitat such as vegetation abundance, turbidity, the nature of the substrate, and the speed of the water.

The vegetation abundance was estimated as the coverage rate of each species. The turbidity of the water was classified by observing the water through a test-glass with a black cross on the bottom. The more water there was in turbid, the less of the cross would be visible. The substratum in the breeding sites was described as rocky, muddy, and sandy. The speed of the water was calculated using the following formula: S = D/t (with S, the mean speed in m/s; D, the distance between the two points in meters, and t, the time in seconds).

Two trained field collectors carried out the sampling using standard snail scoops or occasionally just their hands to collect directly snails on supports (aquatic vegetation, wastes, etc.) [12]. The same collectors scooped for snails in all areas to achieve some level of standardized sampling effort. Sampling time was fixed to 15 minutes per location and was performed between 08:30 h and 10:30 h. Each sampling area per location was approximately 10 m^2^, and lengths of 10 m along streams were used. Snails deeper than one meter in the water were not collected. After each collection, snails from each site were appropriately labeled and transported in separate perforated plastic containers to the Department of Epidemiology and Infectious Diseases (DEAP) of the Faculty of Medicine at the University of Technique and Technologies of Bamako (USTTB) where they were processed. The snail density was expressed as the number of snails captured per collector during the 15 minutes at each site. In our case, where there were two collectors, the total number of snails captured by each collector was divided by two (the number of collectors). At the laboratory, snails were identified to species level based on shell morphological characteristics using standard keys (*Bulinus truncatus*, *B. globosus*, and *Biomphalaria pfeifferi*) [13, 14]. Then snails were rinsed and placed individually in 24-well culture plates containing 1 ml of clear, filtered water (same source as site of collection) and exposed to artificial light for 1–2 h to induce cercarial shedding. The infection was diagnosed and the foci of schistosomiasis transmission were taken to be the breeding sites that presented infected snails [15]. The wells of the plates were then examined for the presence of cercariae under a dissecting microscope. Snails that did not shed cercariae on the first exposure were re-exposed the next day. Following this, the snails were smashed between two glass slides in order to verify the presence of *S. haematobium* cercariae or sporocyst. Bifurcate cercariae were used to indicate that the cercariae were of mammalian origin. To determine the snails’ natural infection rate, the proportion of the mollusks that were positive for *S. mansoni* or *S. haematobium* in relation to the total number of mollusks examined was calculated.

### Parasitological survey

One stool and urine examination census survey was conducted among the sample of schoolchildren selected inside or near each of the 29 blocks by using the same methodology: All individuals were asked to register and participate voluntarily; after giving their oral consent, each child was given plastic containers to collect their stool and urine samples; the urine samples were collected on one day and the stool samples were collected the following day. The samples (stool and urine) were examined on fields *in situ*. To diagnose the number of cases of schistosomiasis and the parasitic load of the parasitized individuals, feces and urine samples were parasitologically diagnosed by means of the Kato-Katz [16] and filtration methods, respectively, with one sample from each participant. The prevalence rate of schistosomiasis in the blocks/schools was defined as the number of positive individuals per block/school per total number of individuals examined per block/school and multiplied by 100. *S. haematobium* intensity was classified into three categories: i) no egg; ii) slight (1–49 eggs per 10 ml of urine); and iii) heavy (≥50 eggs per 10 ml of urine). *S. mansoni* intensity was classified into four classes: *i*) no egg; *ii*) 1–99 epg (eggs per gram of stool); *iii*) 100–399 epg; and *iv*) ≥ 400 epg [17].

### Collection of sociodemographic data

Sociodemographic data including gender, age, and parents’ occupations were obtained using a structured questionnaire. Children were invited to answer questions separately. The distance between the school and the snail breeding sites was calculated as a risk factor of schistosomiasis transmission.

### Statistical analysis

Data were double entered using Access and Prevalence, and the intensity of infection with 95% confidence intervals were calculated using SPSS (IBM, version 19). Differences in proportions were tested using the chi-square test, either for trend or for independence as appropriate, with a risk of 0.05. A multivariate analysis identified the following factors associated with the infection: Niger River side (left *vs*. right); gender (male *vs*. female); age (from 6 to 15 years); parents’ occupations (civil servants, traders vs. working classes, drivers, cleaners, etc.); and distance between the school and snail breeding sites (≤100 m and ≥500 m). In the initial logistic model, all variables associated with the infections in the univariate analysis were included, with p < 0.20. Variables with statistically significant associations (p < 0.05) with *S. haematobium* infection were kept in the final model.

### Ethical approval

The proposal was reviewed and approved by the Institutional Review Board (IRB) of the Faculty of Medicine, Pharmacy and Dentistry, University of Bamako. Community consent was obtained before starting the study. Each study participant signed an assent form.

## Results

### Environmental characteristics

In the Niger River, the substratum is mainly rocky, sandy, or muddy. The lack of vegetation throughout the riverbed has led to the rise of the water speed (35–40 m/s). In contrast, in the tributary, Woyowayanko, the substratum was mainly rocky with a lot of vegetation, which is associated with snail breeding sites (algae and other immerged and submerged species). The speed of water in the stream was about 30 m/s. On the borders of the tributaries, there are some trees (*Mangifera indica*, *Khaya senegalensis*, and *Acacia senegalensis*), which contribute to the humidity around the snail breeding sites especially favorable for *Biomphalaria* growth. According to the turbidity, water was more turbid in the Niger River than in the streams except after a rainy period. The speed of water varied from 11 m/s to 28 m/s in the stream (Woyowayanko), whereas it was higher (35–45 m/s) in the River.

### Malacological survey

The Niger River and its tributaries (streams) were surveyed for *S. haematobium* and *S. mansoni* intermediate hosts from October 2011 to February 2012, and snails were collected using a scoop. The snails have also collected occasionally using big nippers according to the nature of water collection. *Bulinus truncatus*, *B. globosus*, and *Biomphalaria pfeifferi* were collected from both water bodies (see Table 1). They were found attached to leaves falling from surrounding trees, stones, decaying wood, plastic, etc. Other snail species, which are not involved in human schistosome transmission (*Bulinus forskalii* and *Lymnaea natalensis*, *Bellamya unicolor* or *Lanistes varicus*), were also collected from the breeding sites.

**Table 1:** **Distribution of schistosomiasis host snails collected from October 2011 to February 2012 in Bamako**

Months snails	October 2011	November 2011	December 2011	January 2012	February 2012	Total
Pulmonates						
*Bulinus truncatus*	51	36	108	93	36	324
*Bulinus globosus*	02	0	13	0	0	15
*Biomphalaria pfeifferi*	12	42	78	39	18	189
*Bulinus forskalii*	41	33	11	9	5	99
*Lymnaea natalensis*	66	73	49	18	23	229
Operculates						
*Bellamya unicolor*	17	25	12	6	19	79
*Lanistes varicus*	8	3	1	0	2	14

The host snails, *Bulinus truncatus*, *B. globosus*, and *Biomphalaria pfeifferi*, were found with high density in December with 108, 13, and 78 snails collected per person per 15 minutes, respectively (see Table 1).

**Figure 3:**
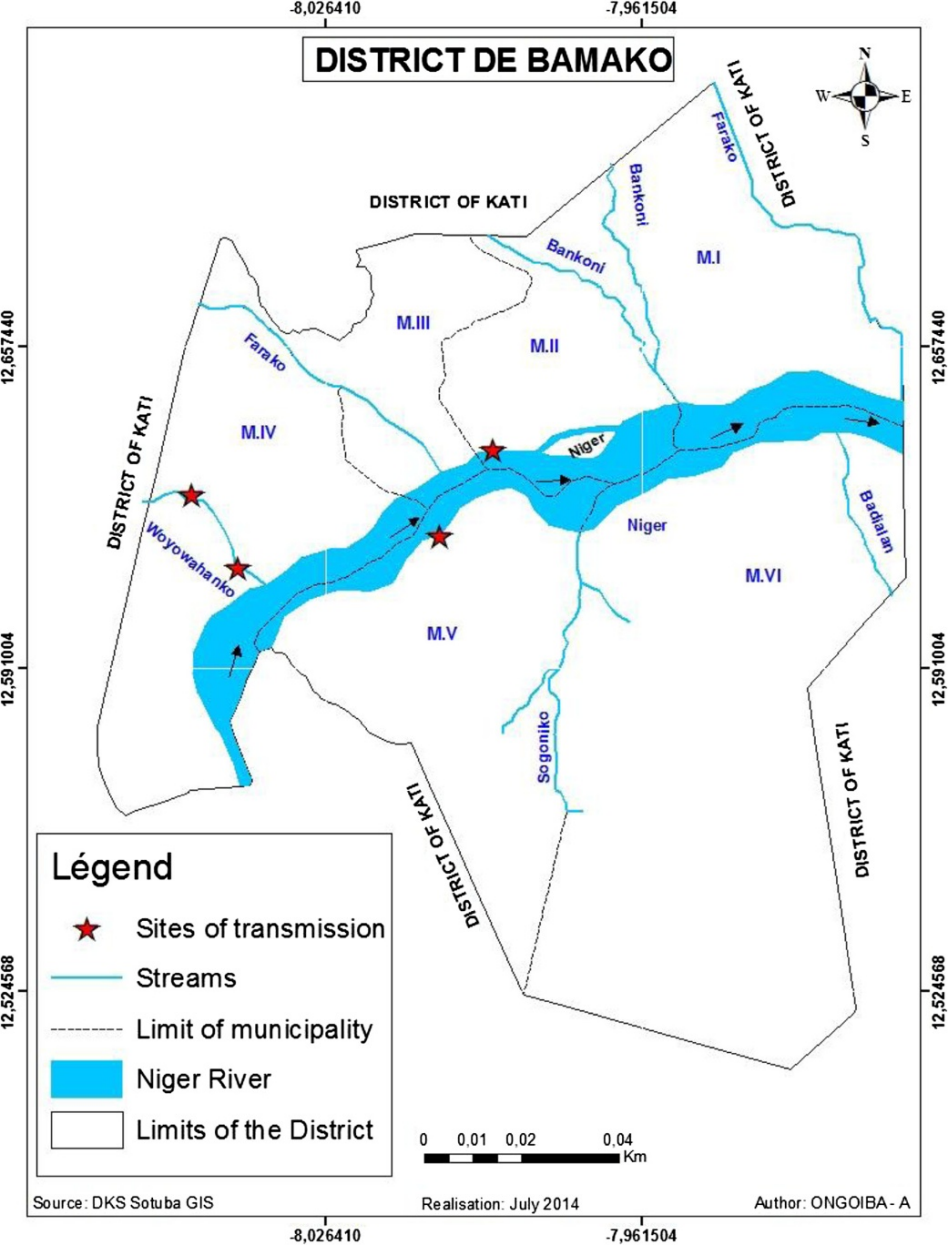
**Map of the district of Bamako: Location of host breeding sites along the Niger River and temporary streams.** MI-MVI: Municipalities the same as CI-CVI.

The foci of transmission were described on the Niger River and the streams (see Figure 3). *B. truncatus* infected with *S. haematobium* was found at two points on the tributary of Woyowayanko and two points on each side of the Niger River in CII (see Figure 3). In contrast, *Biomphalaria pfeifferi* infected with *S. mansoni* was found at two points of Woyowayanko. No infected *B. globosus* were found during the survey.

**Figure 4:**
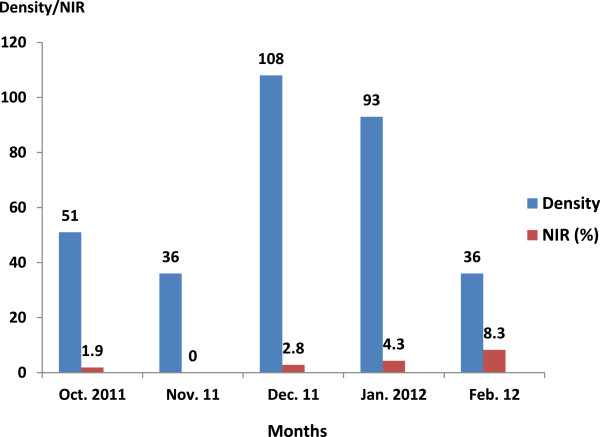
**Density and natural infection rates (NIR) of** ***Bulinus truncatus*** **in the host breeding sites of the district de Bamako.**

**Figure 5:**
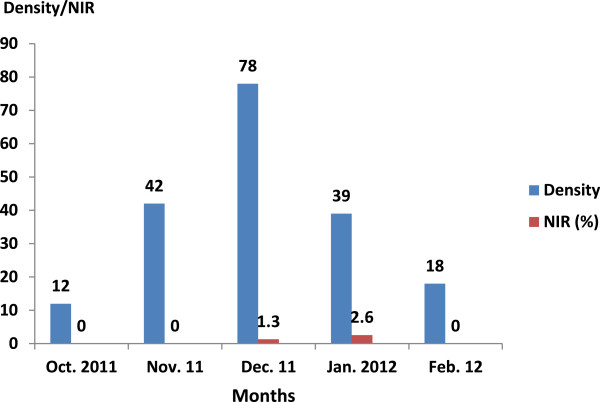
**Density and natural infection rates (NIR) of** ***Biomphalaria pfeifferi*** **in the host breeding sites of the district de Bamako.**

The frequency and the infection rates of *Bulinus truncatus* and *Biomphalaria pfeifferi* are shown in Figures 4 and 5. *B. truncatus* infected with *S. haematobium* was found from October to February, except for in November. The prevalence of schistosome infection in *Bulinus truncatus* was found in February. *B. pfeifferi* infected with *S. mansoni* was found only in December and January (see Figure 5).

### Parasitological survey

From the 29 schools surveyed, the prevalence rates of *S. haematobium*, *S. mansoni*, and those doubly infected were 14.7% (259/1,761), 1.5% (22/1,491), and 1.0% (15/1,459), respectively. Overall, 2.4% (42/1,761) were heavily infected with *S. haematobium*. For *S. mansoni*, only 0.5% (7/1,491) of positive children excreted 400 epg or more.

The initial logistic model included the following variables: the River’s sides (p < 10^-4^), the age (p < 10^-4^), the gender (p = 0.14), the parents’ occupations (p < 10^-4^), and the distance between the school and the snail breeding sites (p < 10^-4^) (see Table 2). Except for gender, the prevalence of *S. haematobium* varied significantly according to the other variables: the schoolchildren on the left side of the River were significantly more infected than those on the right side; children aged 11–15 years were more infected than those aged 6–10 years; the children whose parents were non officials were more exposed; and the prevalence of the infection was higher in the schools located nearer to the snail breeding sites compared to those located further from the snail habitats. Results of the multivariate analysis showing the relationship between *S. haematobium* infection and sociodemographic indicators are shown in Table 3. The most important factors associated with the infection were parents’ occupations (officials vs. non officials: OR = 7.647; CI95%: 2.406–24.305) and the distance between the school and the snail breeding sites (≤100 m vs. ≥500 m: OR = 3.67; CI95%: 2.765–4.889).

**Table 2:** **Univariate analysis of selected environmental and sociodemographic variables and the** ***S. haematobium*** **infection in Bamako, January, 2011**

***Schistosoma haematobium/*** Environmental and Sociodemographic variables	Total	Positive	Prevalence	p
River sides				
Right	592	56	9.5	
Left	1,169	203	17.4	0.000
Total	1,761	259	14.7	
Age group (years)				
6-10	1,462	184	12.6	
11-15	299	75	25.1	0.000
Total	1,761	259	14.7	
Gender				
Male	902	141	15.6	
Female	859	118	13.7	0.146
Total	1761	259	14.7	
Parents’ occupations				
Officials*	174	3	1.7	
Non officials**	1,587	256	16.1	0.000
Total	1,761	259	14.7	
Distance between schools and the snails’ breeding sites				
≤100 m	658	171	26.0	
>500 m	1,103	88	8.0	0,000
Total	1,761	259	14.7	

*: Civil servants, traders.

**: Working classes, drivers, cleaners, etc.

**Table 3:** **Multivariate analysis of selected environmental and sociodemographic variables and the** ***S. haematobium*** **infection in Bamako, January 2011**

Sociodemographic/Variables	N*	Positive	%	p	OR	95% IC
Niger River sides						
Right	592	56	9.5	0,000	1.909	1.376-2.648
Left	1,169	203	17.4	0,000		
Age group (years)						
6-10	1,462	184	12.6			
11-15	299	75	25.1	0.000	0.481	0.349-2.664
Parents’ occupations						
Officials+	174	3	1.7			
Non officials++	1,587	256	16.1	0.001	7.647	2.406-24.305
Distance between schools and the snails’ breeding sites						
≤100 m	658	171	26.0			
≥500 m	1,103	88	8.0	0.000	3.677	2.765-4.889

N: Total number of individuals examined.

+: Civil servants, traders.

++: Working classes, drivers, cleaners, etc.

**Figure 6:**
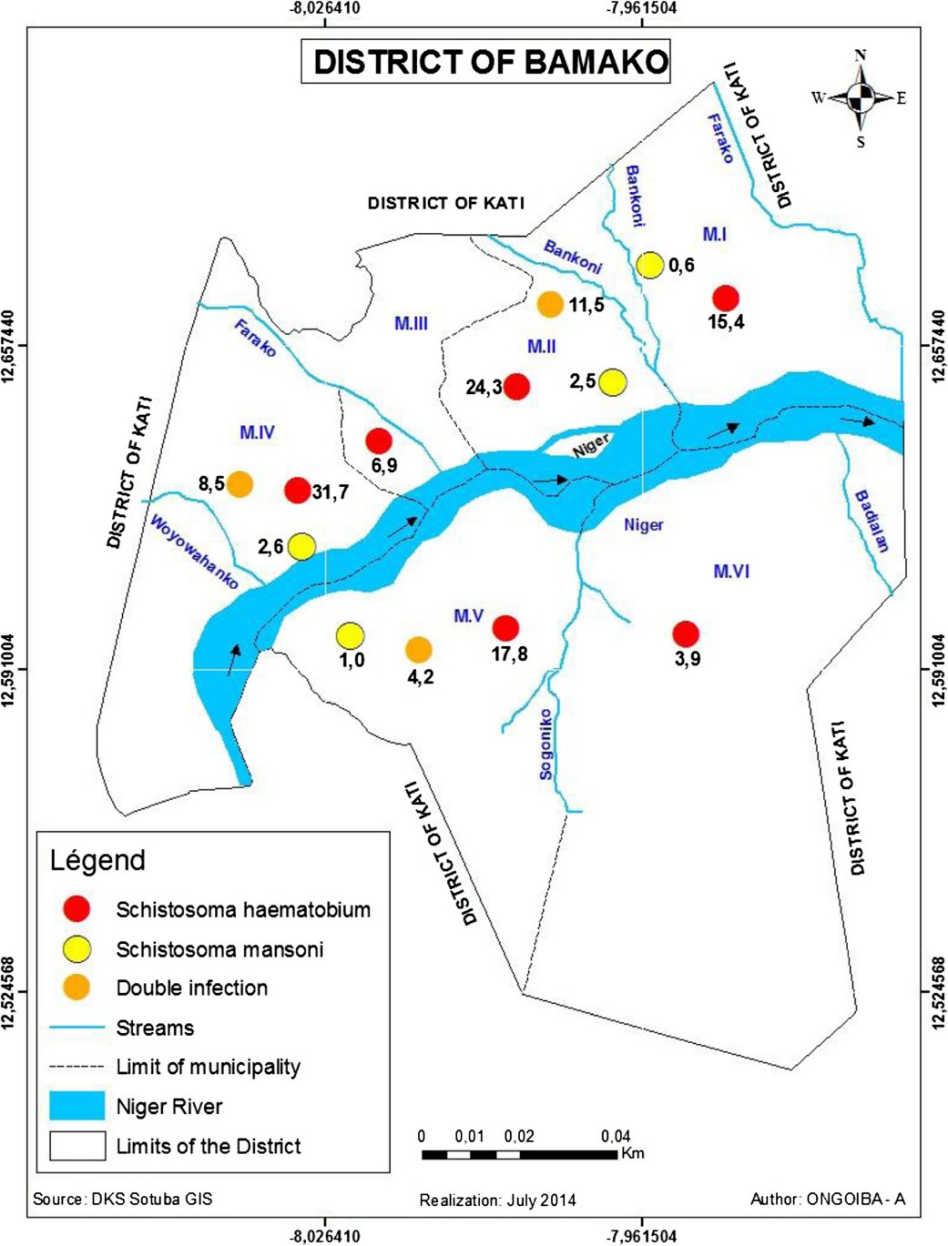
**Distribution of** ***S. haematobium*** **,** ***S. mansoni*** **and double infection in the district of Bamako, November 2011.**

After the distribution of infection through the district was mapped (see Figure 6), it was evident that *S. haematobium* was present in all six municipalities. Its prevalence varied from 31.7% in the M-IV to 3.9% in the M-VI. *S. mansoni* was recorded in four districts where the prevalence was higher (2.6%) in the M-IV and lower (0.6%) in the M-I. Double infection was reported in the M-II (11.5%), -IV (8.5%), and -V (4.2%) districts.

**Figure 7:**
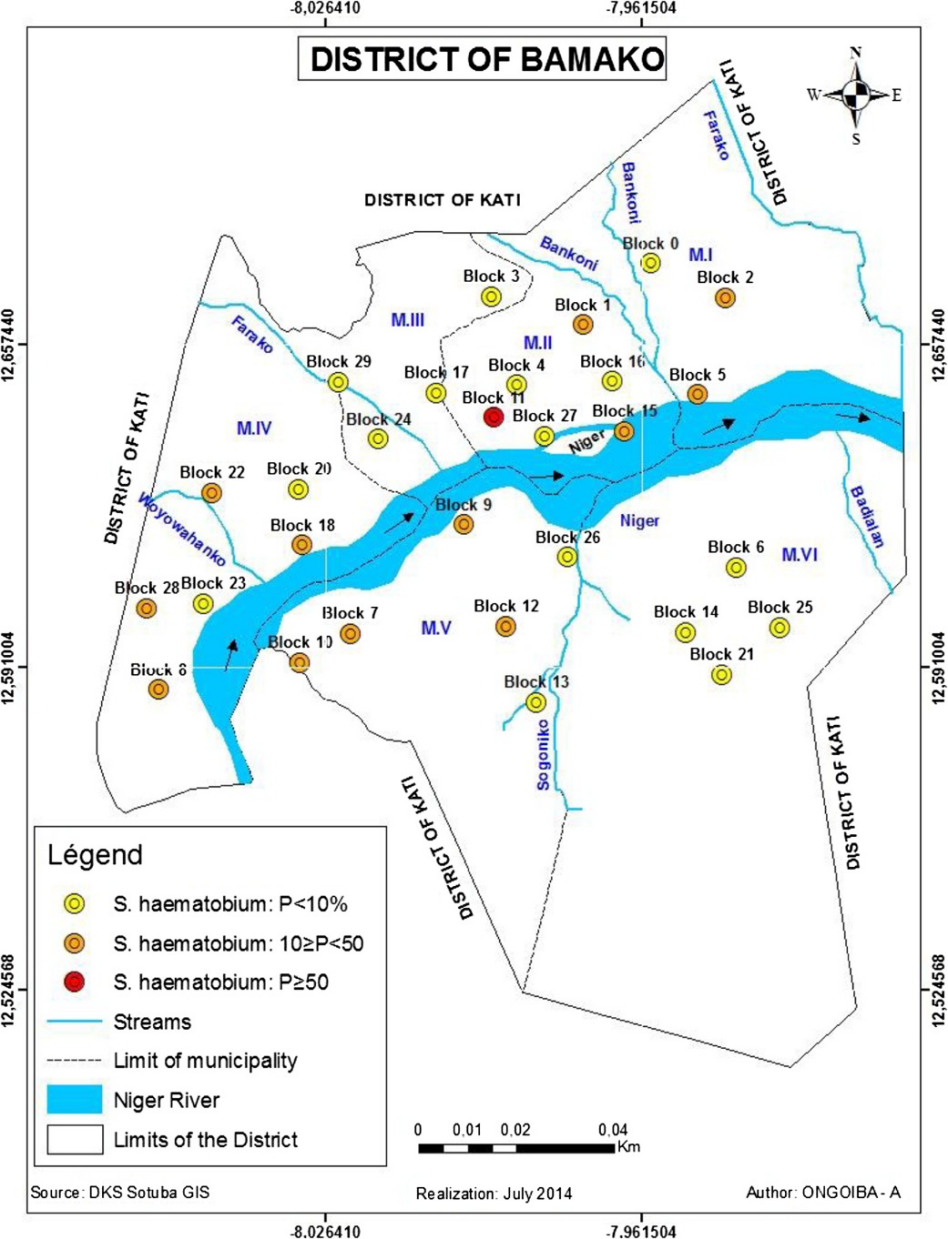
**Endemicity of** ***S. haematobium*** **in the schools communities of the district of Bamako, November 2011.**

According to the endemicity of the disease throughout the schools, *S. haematobium* was hyperendemic (P ≥ 50%) in one school of the municipality -II (see Figure 7), mesoendemic (10% ≥ P < 50%) in 12, and hypoendemic in 16 (P < 10%).

## Discussion

From October 2011 to February 2012, we captured three intermediate hosts: *Bulinus truncatus*, *B. globosus*, and *Biomphalaria pfeifferi*. During the snail survey, physical characteristics of the water bodies showed that the Woyowayanko stream was moderately turbid, and covered by large amounts of weeds, algae (70% of coverage in some sites), and other garbage such as plastic, clothes, and fallen leaves. It was small and slow flowing (less than 0.6 m/s). The substratum of the water body was rocky (90–100% of coverage of the sites) and sandy (10% of coverage in one site). On the other hand, the Niger River was large, fast flowing, clear, and lacked any vegetation, weeds, or algae. The substratum of the water was sandy and muddy. The snail survey showed a bigger abundance of snails (*B. truncatus* and *B. pfeifferi*) in the Woyowayanko stream and relatively few in the Niger River. The reason for the observed difference in the amount of snails in the two water bodies could be explained by the fact that the stream is slow flowing and is abundantly covered with aquatic weeds, whereas the River is not. It has been reported that small rivers with flow rates of 10–30 m/s, with slight turbidity, abundant vegetation at the edge, and that are muddy are potentially favorable habitats for pulmonates snails that could be involved in human schistosome infection [18].

The presence of three intermediate host snail species in Bamako, *B. truncatus*, *B. globosus*, and *B. pfeifferi*, pose a permanent threat to schistosomiasis transmission, as two species were found to be infected by the trematode. This requires attention by health agents because, despite the low prevalence rates of the disease, there is a high risk of schistosomiasis expansion. Other factors contributing to this expansion include poor sewage systems, toilet water thrown in water collections where snails breed, and leisure and domestic activities associated with water contact.

All species involved in human schistosome transmission were described previously in many foci including the rice-irrigated area of Office du Niger, around the Sélingué and Manantali dams, in the Dogon country, and in Bamako [19–21]. In our study, the prevalence of the *S. mansoni* infection in *Biomphalaria pfeifferi* was 2.6% in January. This prevalence was lower compared to the 16.9% of the prevalence of the same species in February, but higher than the 0.027% in April in Sanja area, Amhara region, Ethiopia [22].

The endemicity of schistosomiasis now presents a dual picture. In some regions (North Africa, Asia, Caribbean, the Middle East, and Latin America), many programs have been successful in reducing the mortality, morbidity, and transmission, yet schistosomiasis remains a major cause of mortality and morbidity in a number of countries, especially those in Sub-Saharan Africa [23]. For the establishment of schistosomiasis in new transmission foci, the ecologies and environmental conditions, appropriate aquatic snail intermediate hosts, and the human definitive host must converge in space and time in suitable water bodies. In the case of Bamako, the endemic focus might have been established as a result of population movement into the suburban area from other endemic localities (regions of Ségou, Kayes, Mopti, and Koulikoro) [24, 25].

According to the WHO (2000), migrants contribute to the transmission and spread of schistosomiasis in at least three ways: (i) by introducing the parasite into non-endemic areas, (ii) by creating habitats for snail intermediate hosts and water contact points in the areas where they settle, and (iii) by direct moves in which infected people migrate to areas where schistosomiasis has been controlled or eradicated [26]. In any of these cases, the settlement of populations in suburban agglomerations like in Bamako and the lack of sanitation and basic infrastructures result in fecal contamination of aquatic environments, with consequent infection of intermediate hosts and emergence of new foci of schistosomiasis transmission. Concerning the bio-ecological aspect, favorable environmental conditions were detected for vector reproduction (habitat) and parasite survival (pollution of water collections by stool and urine residues thrown in the River and streams) on the left side of the River as compared to the right side.

Our study revealed that there is *S. haematobium* and *S. mansoni* transmission in the urban area of Bamako, with the occurrence of infection at 14.7% (259/1,761) and 1.5% (22/1,491), respectively. The predominance of *S. haematobium* compared to *S. mansoni* has been reported before in Mali [3, 27]. Adaption of *Bulinus truncatus*, the major host snail of *S. haematobium* to a broad range of environmental conditions such as temperature, rainfall, and standing and flowing water bodies could be the reason for the widespread *S. haematobium* infection [21].

The factors, which were predictive of infection in our study, were the River side, age of child, the sociodemographic status of the parents, and the location of schools in relation to snail breeding sites. Children whose parents were workers were seven times more likely to be infected than children whose parents were civil servants (p < 0.001). The distance between the school where a child lives and the snail-colonized water source was another important exposure risk factor of infection. Children whose schools were located less than 100 m from the stream or River were three times more likely to be infected. According to the diversity of the urban area, children in the peripheral municipalities were likely to be more infected due to the presence and use of Niger River and its tributaries. The results are in agreement with studies conducted in Kenya and Malawi [28, 29]. In studies conducted in Brazil and Zambia, significant risk factors for infection were the male gender [odds ratio (OR) 2.42], and the ages of 9–12 years or 13–17 years (OR 3.33 and 3.26, respectively), compared to 5–8 year olds [30, 31]. In Nigeria, human urinary schistosomiasis appears to be highly endemic in peri-urban/rural areas and closely associated with poverty (a low family income, not living with biological parents). Literacy of the family head was, however, a protective factor [32].

Independently to the vicinity of water bodies, poor sanitation around water sources is a major cause of infection: the prevalence rates of *S. haematobium* and *S. mansoni* were 46.7% and 28.2%, respectively, in 1997 around the Farako stream, one of the Niger River tributaries (see Figure 3). During this period, Farako served as a source of water for laundering, bathing, and other domestic and recreational activities. Most of the quarter dwellers, and especially children, excreted their feces throughout the stream, near the water bodies. The stream became a dumping ground propitious for snail growing. But, since 2010, the stream is free from snails and the prevalence rates in the schools bordering it decreased to zero. In general, with the implementation of mass chemotherapy with praziquantel since 2005, prevalence rates and intensities decreased significantly. However, infection persists due to poor sanitation along the other water bodies (streams and River).

## Conclusion

The finding of *S. haematobium* and *S. mansoni* infected children, and the collection of *Bulinus truncatus* and *B. pfeifferi* infected with schistosome cercariae all confirmed the transmission of schistosomiasis in Bamako. Therefore, based on our findings, appropriate integrated control measures (annual mass drug administration with praziquantel for schoolchildren living along the Niger River and streams, and sanitation of water bodies) need to be introduced to control the transmission of this disease in the study area.

## Electronic supplementary material

Additional file 1: **Multilingual abstracts in the six official working languages of the United Nations.** (PDF 285 KB)

## Abbreviations

ACI 2000: Agence de Cession Immobilière
B *Bulinus*: *Bulinus forskalii*
M-I–M-VI: Mun icipalities
CI: Confidence interval
DEAP: Department of Epidemiology and Infectious Diseases
GPS: Global positioning system
IBM: International Business Machines Corporation
ID: Identification number
IRB: Institutional Review Board
NASA: National Aeronautics and Space Administration
SNIR: Schistosome natural infection rate
OR: Odds ratio
P: Prevalence
*p*: Probability
SPOT: Satellite Pour l’Observation de la Terre
SPSS: Statistical Package for the Social science
vs: *versus*
WHO: World Health Organization.
