# Supplementary material for: Urban schistosomiasis and associated determinant factors among school children in Bamako, Mali, West Africa
Source: Infect Dis Poverty. 2015 Jan 29;4:4. doi: 10.1186/2049-9957-4-4 (PMC4429506; doi:10.1186/2049-9957-4-4)
Supplement: Supplementary file 1 — Additional file 1: Multilingual abstracts in the six official working languages of the United Nations. (PDF 285 KB) [file 40249_2014_96_MOESM1_ESM.pdf]

Translation of the abstract into the six official working languages of the United Nations

مرض البلهارسيا في المناطق الحضرية والعوامل المحددة المرتبطة به بين أطفال المدارس في باماكو، مالي، غرب أفريقيا

عبدالله دابو، أداما ز ديارا، فانيسا ماشولت، عثماني توري، ديارا سيرا نيامبيلي، عبدالله كانتي، عبدالله أونجويبا، أوجوبارا دومبو

الملخص

الخلفية: يوصف مرض البلهارسيا تقليدياً كمرض ريفي يحدث في مناطق ذات ظروف صرف صحي سيئة. مع ذلك، خلال العقود الأخيرة، كان هناك تمدد لبؤر مرض البلهارسيا نحو مناطق حضرية متجهة إلى تحضر سريع ومضطرب. في باماكو، مالي، تأثير التغيرات البيئية على الأمراض المنقولة بناقل مثل مرض البلهارسيا غير معروفة جيداً. سعت هذه الدراسة إلى التعرف على وجود بؤر انتقال مرض البلهارسيا في باماكو. وباستخدام هذا المنظور، فإننا نهدف لوصف عوامل الخطر لتوطن وإعالة مرض البلهارسيا.

طرق العمل: تم إجراء دراسة عرضية في ست بلديات (وحدات إدارية) في باماكو.وتم الحصول على المعلومات البيئية من أقمار صناعية تراقب الأرض من أجل تعظيم التباينات البيئية. وقد تم تحديد تسعة وعشرين كتلة بمساحة 200 متر × 200 متر. تم اختيار مدرسة داخل أو بالقرب من كل كتلة لفحص عينات البول والبراز. وكانت مجموعة الدراسة من تلاميذ المدارس الذين تتراوح أعمارهم بين ثمانية و 15 سنة. تم استخدام أسلوب كاتو-كاتز والترشيح للبحث عن بويضات البلهارسيا المعوية والبلهارسيا البولية في البراز والبول، على التوالي. تم إعداد خرائط بأماكن المدارس ومواقع تكاثر القواقع. تم إجراء أربع دراسات مسحية للرخويات بين أكتوبر وفبراير. تم استخدام التحليل ثنائي المتغير لتحديد المنبئات المستقلة للإصابة بمرض البلهارسيا.

النتائج: كان معدل انتشار البلهارسيا البولية (n=1,761) 14,7% ومعدل البلهارسيا المعوية (n=1,491) 1,5%. وعموماً، كان النوع البولي مستوطناً في 76.6% من المدارس. تفاوتت الإصابة بشكل كبير بين البلديات (*P<0.001*). وكان أيضاً أكثر انتشاراً على الجانب الأيسر لنهر النيجر من الجانب الأيمن (17,4% مقابل 9,5% على التوالي، *p<0.001*). القرب من مواقع تكاثر القواقع (النتيجة العامة = 3,677؛ 95% مدى الثقة [4,889-2,765]؛ *P<10⁻³*) و مهن الآباء (النتيجة العامة = 7,647؛ 95% مدى الثقة [24,405-2,406]؛ *P<0.001*) كانت هي أكثر عوامل الخطر الهامة ارتباطاً بالتعرض للإصابة بالبلهارسيا البولية. كانت قواقع البيومفلاريا فيفري، والبولينس ترونكاتس والبولينس جلوبوسيس هي العائل الوسيط الظاهر. معدلات الإصابة الطبيعية بالبلهارسيا (SNIRs)، التي كانت منخفضة أو معدومة خلال شهري أكتوبر ونوفمبر، ارتفعت إلى 2,8% خلال يناير و 8,3% خلال فبراير بالنسبة للبيومفلاريا فيفري والبولينس ترونكاتس، على التوالي.

الاستنتاج: تشير النتائج إلى أن هناك خطر انتقال شديد لمرض البلهارسيا في باماكو. هناك حاجة لاستخدام إجراءات رقابية متكاملة ومناسبة للسيطرة على انتقال هذا المرض في منطقة الدراسة.

Translated from English version into Arabic by Saher Salama, through

TRANSLATORS
WITHOUT BORDERS

西非马里首都巴马科的城市血吸虫病及其在学龄儿童间的相关作用因素研究

Abdoulaye Dabo, Adama Z Diarra, Vanessa Machault, Ousmane Touré, Diarra Sira Niambélé, Abdoulaye Kanté, Abdoulaye Ongoiba, Ogobara Doumbo

摘要

引言：血吸虫病通常被说成是农村疾病，在卫生状况较差地区发生。但是，近几十年来，血吸虫病疫点随着快速而混乱的城镇化形势不断扩大。在马里首都巴马科，环境变化对诸如血吸虫病这样的媒传病影响还了解。本研究旨在鉴定巴马科存在的血吸虫病传播热点地区，并从这个角度分析血吸虫病地方性流行和维持的风险因素。

方法：在巴马科的6个市镇开展横断面研究，通过地球观测卫星获得环境信息以期最大程度地显示生态差别。以9个“200米x200米”的街区为目标，在每个街区内或离得最近处选择一所学校，进行尿样和粪样检测。研究组年龄为8-15岁。使用改良加藤法和过滤法分别检测粪样和尿样的曼氏血吸虫和埃及血吸虫卵。同时做好学校和螺孳生地的地理坐标。还在十月份到二月份之间开展4次贝类调查。最后使用双变量分析鉴定感染血吸虫病的独立危险因素。
结果：埃及血吸虫病和曼氏血吸虫病的患病率分别是14.7%（n=1,761）和1.5%（n=1,491）。整体上，有76.65%的学校有埃及血吸虫病地方性流行。各市镇的感染情况存在统计学差异（*P<0.001*）。尼日尔河左岸患病率（17.4%）高于右岸（9.5% ）（*p<0.001*）。靠近螺孳生地（OR=3.677；95% IC [2.765–4.889]；$P<10^{-3}$）和父母职业（OR=7.647；95%IC [2.406–24.305]；*P<0.001*）是埃及血吸虫感染最重要的危险因素。还捕获到3种中间宿主螺类（*Biomphalaria pfeifferi*，*Bulinus truncatus*和*B. globosus*）。其中，*B. pfeifferi*和*B. truncatus*的血吸虫自然感染率从十月份和十一月份的很低或零水平上升到一月份的2.8%和二月份的8.3%。
结论：本研究发现巴马科是血吸虫病的高风险传播地区，应采用合理的综合控制措施来控制本研究地区的血吸虫病传播。

Translated from English version into Chinese by Yin Jian-hai, through

CHINESE CENTER FOR DISEASE CONTROL AND PREVENTION
NATIONAL INSTITUTE OF PARASITIC DISEASES

Schistosomiase urbaine et facteurs déterminants associés parmi les enfants scolarisés à Bamako au Mali (Afrique de l'Ouest)

Abdoulaye Dabo, Adama Z Diarra, Vanessa Machault, Ousmane Touré, Diarra Sira Niambélé, Abdoulaye Kanté, Abdoulaye Ongoiba, Ogobara Doumbo

Résumé

Contexte : La schistosomiase est généralement considérée comme une maladie rurale, présente dans les zones où les conditions sanitaires sont insatisfaisantes. Toutefois, au cours des dernières décennies, les foyers de schistosomiase se sont déplacés vers les zones des villes soumises à une urbanisation à la fois rapide et désordonnée. À Bamako au Mali, l'impact des changements environnementaux sur les maladies vectorielles telles que la schistosomiase est encore mal connu. L'objectif de cette étude était d'identifier la présence de foyers de transmission de la schistosomiase à Bamako. Dans cette perspective, nous voulions décrire les facteurs de risque d'une endémisation et d'une persistance de la schistosomiase.
Méthodes: Une étude transversale a été menée dans les six communes de Bamako. Pour optimiser les contrastes écologiques, les données environnementales ont été recueillies depuis des satellites d'observation de la Terre. Nous avons identifié vingt-neuf blocs de 200 m x 200 m. À l'intérieur ou au plus près de chaque bloc, nous avons sélectionné une école pour procéder à l'analyse d'échantillons d'urine et de selles. La cohorte de l'étude était composée d'enfants scolarisés âgés de 8 à 15 ans. Pour la recherche des oeufs de *Schistosoma mansoni* et de *S. haematobium* respectivement dans les matières fécales et dans l'urine, nous avons eu recours à la technique de Kato-Katz et à un système de filtration. Nous avons géoréférencé les écoles et les sites d'élevage d'escargots. Entre octobre et février, nous avons mené quatre études malacologiques. Pour identifier les variables indépendantes prédictives d'une contamination par la schistosomiase, nous avons utilisé l'analyse bivariée.

Résultats: Le taux de prévalence de *S. haematobium* était de 14,7 % (n = 1761) et celui de *S. mansoni* de 1,5 % (n = 1491). Globalement, la forme urinaire était endémique dans 76,6 % des écoles. Le taux de contamination était très variable en fonction des communes ($P < 0,001$). Le taux de prévalence était également plus élevé sur la rive gauche du fleuve Niger que sur la rive droite (17,4 % contre 9,5 % ; $p < 0,001$). La proximité avec les lieux d'élevage des escargots (OR=3677 ; 95 % IC [2765–4889]; $P<10^{-3}$) et le métier des parents (OR=7647; 95 % IC [2406–24 305], $P < 0,001$) constituaient les principaux facteurs de risque associés à l'exposition à *S. haematobium*. Les hôtes intermédiaires capturés étaient *Biomphalaria pfeifferi*, *Bulinus truncatus* et *B. globosus*. Les taux de contamination naturelle par schistosoma qui étaient bas ou inexistants en octobre et novembre, ont atteint 2,8 % en janvier et 8,3 % en février pour *B. pfeifferi* et *B. truncatus* respectivement.
Conclusion: Nos résultats montrent qu'il existe un risque élevé de transmission de la schistosomiase à Bamako. Il faut introduire des mesures de contrôle intégré appropriées pour contrôler la transmission de la maladie dans la zone étudiée.

Translated from English version into French by Password Europe, through

TRANSLATORS
WITHOUT BORDERS

Городской шистосомоз и сопутствующие определяющие факторы среди школьников в Бамако, Мали, Западная Африка

Абдулайе Дабо, Адама Z Диарра, Ванесса Машо, Усман Туре, Диарра Sira Ниамбеле, Абдулайе Канте, Абдулайе Онгоиба, Огобара Думбо

Синопсис
Справочная информация: Шистомоз классически описывается как сельской болезнь, которая возникает в районах с плохими санитарными условиями. Тем не менее, в последние десятилетия наблюдается расширение очагов шистосомоза в городских районах с быстрой и неупорядоченной урбанизацией. В Бамако, Мали, влияние изменений окружающей среды на такие трансмиссивные заболевания, как шистосомоз, не известно. Это исследование ставило целью определить наличие горячих точек заражения шистосомозом в Бамако. С этой точки зрения мы стремились описать факторы риска в эндемизации и сохранении шистосомоза.
Методы: Перекрестное исследование было проведено в шести муниципалитетах Бамако. Экологическая информация была получена из спутников наблюдения Земли в целях максимизации экологических контрастов. Были определены двадцать девять кварталов 200 x 200 м. Мы выбрали школу внутри или недалеко от каждого квартала для отбра образцов мочи и кала на экспертизу. Исследование проводилось на школьниках в возрасте от восьми до 15 лет. Техника Като-Кац и фильтрация были использованы для определения яйцеклеток Schistosoma mansoni и C. haematobium в стуле и моче соответственно. Школы и гнездовья улиток были гео-привязаны. Четыре малакологических исследования были проведены в период с октября по февраль. Двумерный анализ был использован для выявления независимых предикторов заражения шистосомозом.

Результаты: показатель распространенности С. haematobium составил 14,7% (п = 1761), и С. mansoni 1,5% (п = 1491). В целом, мочевая форма является эндемической в 76,6% школ. Инфекции значительно различаются между муниципалитетами (P <0,001). Кроме того, заражение было более распространенным на левом берегу реки Нижер, чем на правом (17,4% против 9,5% соответственно; p <0,001).Близость к гнездовьям улиок (OR = 3,677; 95% IC [2.765-4.889]; P <10-3) и профессиональный статус родителей (OR = 7,647; 95% IC [2.406-24.305]; P <0,001) были наиболее важными факторами риска, связанными с воздействием инфекции С. haematobium. Biomphalaria pfeifferi, Bulinus truncatus, и B. globosus были промежуточными хозяевами. Естественный уровень инфекции шизостомы (SNIRs), который были низким или нулевым в октябре и ноябре, вырос до 2,8% в январе и 8,3% в феврале для B. pfeifferi и B. truncatus, соответственно.

Вывод: Наши результаты показывают, что существует высокий риск передачи шистосомоза в Бамако. Необходимо ввести интегрированные меры для остановки распространенния этого заболевания в исследуемой области.

Translated from English version into Russian by Julia Zarubinska-Toepritz, through

TRANSLATORS
WITHOUT BORDERS

La esquistosomiasis en zonas urabanas y los factores determinantes asociados entre niños de edad escolar en Bamako, Mali, África Occidental

Abdoulaye Dabo, Adama Z Diarra, Vanessa Machault, Ousmane Touré, Diarra Sira Niambélé, Abdoulaye Kanté, Abdoulaye Ongoiba, Ogobara Doumbo

Resumen

Antecedentes: la esquitosomiasis suele ser descrita como una enfermedad rural que se manifiesta en zonas en las que existen condiciones sanitarias inadecuadas. No obstante, en décadas recientes ha habido una propagación de esquistosomiasis foci hacia las zonas urbanas que se enfrentan a una urbanización rápida y desordenada. En Bamako, Malí, no se conoce bien el impacto del cambio medioambiental en las enfermedades transmitidas por vectores, como la esquistosomiasis. El objetivo de este estudio era identificar la presencia de focos de transmisión de esquistosomiasis en Bamako. A través de esta perspectiva, buscamos describir los factores de riesgo donde la esquistosomiasis se vuelve endémica y se mantiene.

Métodos: se ha llevado a cabo un estudio transversal en las seis municipalidades (comunas) de Bamako. Se obtuvo información medioambiental de satélites de observación terrestre a fin de maximizar los contrastes ecológicos. Se identificaron veintinueve manzanas de 200 m por 200 m. Elegimos una escuela dentro o lo más próxima posible a una de esas manzanas para tomar muestras de análisis de orina y de materia fecal. La población base fueron niños de edad escolar de entre ocho y 15 años. Se emplearon la técnica Kato-Katz y la filtración para analizar los huevos de *Schistosoma mansoni* y *S. haematobium* en las heces y la orina, respectivamente. Se georeferenciaron las escuelas y los lugares de crianza de caracol. Se realizaron cuatro encuestas malacológicas entre octubre y febrero. Se empleó un análisis bi-variable para identificar los indicadores independientes de una infección de esquitosomiasis.

Resultados: la tasa de predominio de *S. haematobium* fue de un 14,7 % (n=1,761) y la de *S. mansoni* de un 1,5 % (n=1,491). En general, la forma urinaria fue endémica en un 76,6 % de las escuelas. La infección varió de manera considerable entre las municipalidades ($P<0,001$). También fue más predominante en la orilla izquierda del Río Niger que en la derecha (un 17,4 % vs. un 9,5 % respectivamente; $p<0,001$). Los alrededores de los sitios de crianza de caracol (OR=3,677; 95 % IC [2,765–4,889]; $P<10^{-3}$) y las actividades de los padres (OR=7,647; 95%IC [2,406–24,305]; $P<0,001$) fueron los factores de riesgo más importantes asociados con la exposición a la infección por *S. Haematobium*. Los portadores intermedios capturados fueron *Biomphalaria pfeifferi*, *Bulinus truncatus*, y *B. globosus*. Las tasas de infección natural por esquisotoma (SNIR, son sus siglas en inglés), que fueron bajas o nulas en octube y noviembre aumentaron a un 2,8% en enero y un 8,3% en febrero en el caso de *B. pfeifferi* y *B. truncatus*, respectivamente.

Conclusión: nuestros resultados arrojan que existe un alto riesgo de transmisión de esquitosomiasis en Bamako. Se deben introducir medidas integradas para controlar la transmisión de esta enfermedad en la zona de estudio.

Translated from English version into Spanish by Adriana Acevedo, through

TRANSLATORS
WITHOUT BORDERS
